# Treatment, monitoring, and euthanasia in canine diabetes mellitus - a questionnaire-based survey among veterinarians in Norway

**DOI:** 10.1186/s13028-026-00878-1

**Published:** 2026-08-31

**Authors:** Sivert Nerhagen, Esben Østergaard Eriksen, Jo Amundstad, Kristin Paaske Anfinsen

**Affiliations:** 1 Department of Companion Animal Clinical Sciences, Faculty of Veterinary Medicine, Norwegian University of Life Sciences, Oluf Thesensvei 30, 1433 Ås, Norway; 2 Lifecare Veterinary Ltd, Ytrebygdsvegen 215, 5258 Blomsterdalen, Norway; 3 Department of Production Animal Clinical Sciences, Faculty of Veterinary Medicine, Norwegian University of Life Sciences, Elisabeth Stephansens vei 15, 1433 Ås, Norway; 4 Vetlabs AS, Hyllveien 19, 0274 Oslo, Norway

**Keywords:** Canine diabetes mellitus, Continuous glucose monitor, Diabetic monitoring, Euthanasia, Neutering

## Abstract

**Background:**

Canine diabetes mellitus (DM) is a relatively common endocrinopathy, and it requires dedication and commitment from an owner to care for a diabetic pet. This study aimed to describe treatment and monitoring habits, perceived owner and veterinary concerns when treating and monitoring a diabetic dog, as well as triggers for and risk of euthanasia in diabetic dogs among Norwegian veterinarians. A questionnaire was designed and made available online.

**Results:**

A total of 125 veterinarians completed the survey. According to the data collected, the cumulative incidence risk for treatment initiation was 71.6% (95% CI 63.2, 80.6), suggesting that the respondents started most of the diabetic dogs seen in their practice on treatment. The majority of the respondents reported that all female entire diabetic dogs were neutered near the time of diagnosis. Main veterinary treatment concerns identified were difficulties in achieving rapid and adequate control. Main respondent-perceived owner concerns were cost and injecting their diabetic dog. About 60% of respondents had never used a continuous glucose monitor. Of respondents having used such monitors, half had experienced owners changing the insulin dose themselves, without consulting a veterinarian. Nearly one third of the veterinarians recalled that issues related to monitoring contributed directly to euthanasia of a diabetic dog in their care. The estimated one month and one year cumulative risk of euthanasia in dogs started on insulin therapy found in our study were 11.5% (95% CI 8.3, 14.6) and 26.9% (95% CI 22.0, 31.8), respectively. In our study population, respondents working in large cities were more likely to report a high probability of starting treatment than respondents in small cities and rural areas. Respondents in small cities and rural areas were also more likely to report medium and high risk of euthanasia compared to respondents in large cities, respectively. Veterinary-perceived owner triggers for euthanasia were dogs’ quality of life, monitoring, and owners’ lifestyle changes. For the respondents themselves, comorbidities were the leading factor reported for recommending euthanasia.

**Conclusions:**

This questionnaire-based study describes perceived treatment concerns, and reasons for and estimates of euthanasia risk in dogs with DM, based on the experiences of veterinary practitioners working in Norway. The results highlight important aspects of the treatment and monitoring of dogs with DM, as well as triggers for euthanasia. Further investigations into the aspects identified could be useful to improve the overall care for diabetic dogs.

**Supplementary Information:**

The online version contains supplementary material available at https://doi.org/10.1186/s13028-026-00878-1.

## Background

With reported annual incidence risk in the UK of 0.09% in dogs ≥ 3 years, and estimated prevalence between 0.26% and 0.36% in the UK and Australia, canine diabetes mellitus (DM) is a relatively common endocrinopathy [1–4]. Caring for a diabetic companion animal requires dedication and commitment from the owner, including close collaboration with a veterinarian, especially in a newly diagnosed pet. Several aspects of having a dog with DM worry owners: the dog’s quality of life, costs of treatment, having to inject insulin, and changes in the owner’s lifestyle [5, 6]. Over the last few years, monitoring of diabetic dogs and other companion animals has changed with the introduction of continuous glucose monitors (CGM) [7]. These monitors enable easier, more detailed, and near real-time monitoring of a patient’s glycemic control [7, 8], which might result in a lower risk of diabetic complications and improved glycemic control [9]. Most owners responding to a questionnaire assessing home monitoring with CGM, reported perceived improvement of their pets’ diabetic control, and considered their use easier and less painful compared to blood glucose curves (BGC) [10]. However, little is known about the effect of monitoring on the decision to euthanise a diabetic pet, its implication on veterinary and owner communication and cooperation, and potential challenges faced by veterinarians using CGM.

The decision to euthanise a diabetic dog is naturally multifaceted, and different countries have different cultures, views on animal welfare, and prevailing ethical norms [11, 12]. The Big Diabetes Survey was a veterinary-targeted questionnaire that assessed management, including treatment and euthanasia, of diabetic dogs and cats world-wide. The survey identified differences between geographical location and practice types on euthanasia of diabetic animals, however results for Norway alone were not presented. As such, investigating what difficulties veterinarians experience regarding treatment, monitoring, and euthanasia within different geographical locations and demographics is of importance to optimise care.

This questionnaire-based study aimed to describe veterinary-perceived factors affecting the decision of euthanasia of diabetic dogs; Norwegian veterinarians’ own perception of their treatment and monitoring habits, including CGM-use; and how treatment and euthanasia vary with demographics.

## Methods

This study was a questionnaire-based descriptive survey with self-selection sampling.

### Setting and study population

The target population were veterinarians practising in Norway who currently cared for dogs and who had diagnosed at least one dog with DM. The questionnaire was made available at *formstack.com* between 1 December 2023 and 1 December 2024 and was advertised on a web-based veterinary forum consisting of Norwegian veterinarians and veterinary students. In addition, a link to the questionnaire was advertised through the Norwegian Veterinary Association electronic newsletter. The Norwegian Veterinary Association estimates around 950 small animal and 300 mixed practice veterinarians to be registered within the organisation and on the mailing list (C. Tengs, personal communication). Multiple reminders were posted on the web-based veterinary forum, and four CPD presentations on monitoring and management of canine DM were held as advertisement for the questionnaire. The invitation called for all veterinarians caring for dogs in their practice (i.e., small animal practice or mixed practice) who had diagnosed DM in a dog at least once.

### Design of the questionnaire

The majority of the questions were a Norwegian translation of a previously used DM questionnaire in English [6], allowing direct comparison between studies. This previous DM questionnaire was closed-format, consisting primarily of multiple-choice and numeric-response questions (e.g. of 10 dogs newly diagnosed with DM, how many are started on insulin therapy?). Questions regarding intact female dogs and experiences using CGM were added. To ensure that the questions were DM-focused, and to assess any superfluous or confusing questions or answers, a pilot of the questionnaire was completed by six veterinarians. These were working in mixed practice (large and small animals, *n* = 3), small animal general practice (*n* = 2), and in a referral setting (*n* = 1). The pilot participants were asked to give feedback on the questionnaire in interview form or, if they preferred, in writing. Following the pilot, two questions were removed (one regarding euthanasia and one regarding therapy) as the questions were considered already answered in previous questions by four out of six and all pilot participants, respectively. Response categories were also fine-tuned (e.g. words that were interpreted differently between pilot-veterinarians were changed to clarify the meaning) before the questionnaire was made available online. Due to technical errors, one question was omitted from the questionnaire: “out of 10 newly diagnosed dogs with DM, how many do you euthanise at the time of diagnosis”, whilst the response “comorbidities” was omitted from “Often euthanasia is a combination of the factors given in the previous questions, but of these, what is the most important factor for you?”. Subsequently this question was also excluded from the final analyses.

The first question addressed the inclusion criteria of having diagnosed DM in a dog at least once. The questionnaire was subsequently divided into four parts: general DM questions (mainly focusing on treatment and concerns about treatment), monitoring, euthanasia and demographics. There were 14 questions (one with five sub-questions) in the general DM part, 16 about monitoring (16 if using GCM in their practice, seven if not), nine regarding euthanasia, and three on demographics. All questions had a separate ‘other’ option providing the respondents an option for open-ended answers or comment. The ‘other’ option could be left blank. The participants responded anonymously.

### Statistics

Stata SE (18.0 StataCorp LLC, College Station, TX) was used for statistical analysis. Descriptive statistics were used to show the distribution of answers. To assess the logic of responses, locally weighted scatterplot smoothing and Spearman’s rank correlation were applied to assess the relationship between the respondents’ perceived euthanasia risk due to DM and the percentage of dogs respondents would start on treatment, and to assess the relationship between the questions “would you treat your own dog with insulin if it developed DM” where the answers were given on a four-point scale (from definitely not to definitely yes) and “Of the dogs you diagnose, in what percentage do you start treatment?”. Statistical significance was set at *P* < 0.5. Both starting treatment risks and euthanasia risks were categorised into three groups: low (0–29% and 0–30%, respectively), medium (30–69% and 40–60%, respectively) and high (≥ 70% and 70–100%, respectively) based on Question 2 in the treatment section and Question 3 in the euthanasia section.

Three cumulative incidence risks were estimated at dog level: The risk of treatment initiation in diagnosed dogs, the risk of euthanasia after one month and after one year among dogs started on treatment (Questions 3b, 7 and 8 within the treatment section, respectively). These were estimated by weighing each veterinarian’s response according to the reported annual number of diagnosed diabetic dogs (see Eqs. 1, 2, 3, 4). In essence, scorings made by veterinarians seeing many diabetic dogs were given more weight. 95% confidence intervals for these estimates were estimated with bootstrapping using 1000 replicates. The algorithm for transforming scorings of number of dogs (Question 1 within the treatment section) into an actual number of diagnosed dogs is defined in Eq. 1. Generative AI (Microsoft Co-pilot powered by GPT-5) was used to help suggest how Eqs. 1 and 3 should be notated. The authors made the final decision on the notations.

Equation 1: Piecewise function expressing the annual number of diagnosed dogs with DM

1 $$ \alpha \left( \beta \right) = \left\{ {\begin{array}{*{20}c} {0.5~if~\beta \in \left\{ {0;1} \right\}} \\ {2~if~\beta \in \left\{ {1;3} \right\}} \\ {4~if~\beta \in \left\{ {3;5} \right\}} \\ {7.5~if~\beta \in \left\{ {5;10} \right\}} \\ {15~if~\beta \in \left\{ { > 10} \right\}} \\ \end{array} } \right. $$

where α is the annual number of dogs diagnosed with DM by the respondent, and β is the categorical scoring of number of dogs diagnosed. The Cumulative one-month and one-year euthanasia risks in treated diabetic dogs were estimated at dog level using Eq. 2,

Equation 2: Estimator of the cumulative incidence risk (%) of euthanasia

2 $$ \gamma = \frac{{\mathop \sum \nolimits_{{i = 1}}^{n} \alpha _{i} ~\pi _{i} }}{{\mathop \sum \nolimits_{{i = 1}}^{n} \alpha _{i} }}100^{{ - 1}} $$

where γ is the cumulative incidence risk, $\:{\alpha}_{i}$ is α (as defined in Eq. 1) for respondent *i*, and π is the number of dogs out of 10 that were euthanised after one month and one year, respectively, as reported by respondent *i*.

Equation 3 defines the algorithm for transforming the categorical scoring the percentage of dogs where treatment was initiated into an actual percentage of dogs.

3 $$ \omega \left( \varphi \right) = \left\{ {\begin{array}{*{20}c} {5~if~\varphi \in \left\{ {0;10} \right\}} \\ {20~~if~\varphi \in \left\{ {10;30} \right\}} \\ {40~if~\varphi \in \left\{ {30;50} \right\}} \\ {~60~if~\varphi \in \left\{ {50;70} \right\}} \\ {80~if~\varphi \in \left\{ {70;90} \right\}} \\ {~95~if~\varphi \in \left\{ {90;100} \right\}} \\ \end{array} } \right. $$

The risk of treatment initiation after DM has been diagnosed was estimated based on Question 2 in the treatment section using Eq. 4,

Equation 4: Estimator for the risk (%) of treatment initiation

4 $$ \tau = \frac{{\mathop \sum \nolimits_{{i = 1}}^{n} \alpha _{i} ~\omega _{i} }}{{\mathop \sum \nolimits_{{i = 1}}^{n} \alpha _{i} }}~100^{{ - 1}} $$

where $\:\tau\:$ is the risk of treatment initiation, $\:{\alpha}_{i}$ is α (as defined above in Eq. 1) for respondent *i*, and $\:{\omega}_{i}$, is $\:\omega\:\:$(as defined above in Eq. 3) for respondent *i*.

### Ethical approval

As this study was based on a voluntary questionnaire aimed at veterinarians, where participants could not be identified based on the data obtained through the questionnaire, no ethical approval was deemed necessary by the University ethics committee.

## Results

### Description of the study population and data quality

A total of 131 veterinarians responded to the questionnaire; however, six (4.5%) respondents had never diagnosed canine DM, so 125 veterinarians were included in the study. Using the number of e-mail list receivers as denominator, the response rate was 10.5% (*n* = 131/1.250). The geographical distribution of the respondents (based on county) largely corresponded to the estimated percentage distribution of dogs in 10 of 15 counties (AM. Aardal; unpublished data), with one county (Vestland) providing a higher percentage of responses, and four providing lower (Troms, Trøndelag, Buskerud and Akershus). Most respondents worked in urban or suburban environments (78.6%, *n* = 91/122), solely with small animals (79.8%, *n* = 95/119), and diagnosed between one and three dogs with DM per year (68.7%, *n* = 90/125). See Table 1 for demographic data. In total, 54/125 responses were incomplete with 145 missing answers out of 7.277 possible responses.

**Table 1:** Overview of respondent demographics and diabetic dogs diagnosed annually

Variable	Response categories	*n* respondents (%)
Location	Large city (> 100.000 inhabitants)	36 (29.5%)
	Suburbs to a large city	11 (9%)
	Small city (> 5.000 but < 100.000 inhabitants)	44 (36.1%)
	Rural environments	31 (25.4%)
Practice type	Small animal practice	95 (79.8%)
	Mixed practice	23 (19.3%)
	Only seeing dogs out-of-hours	1 (0.8%)
DM diagnoses per year	< 1 1–3 3–5 5–10 > 10	20 (15.2%) * 90 (68.7%) 10 (7.6%) 4 (3.1%) 1 (0.8%) **

* Most respondents stating one dog every two years

** Stating they worked in a charity clinic

The association between perceived euthanasia risk due to DM and the percentage of dogs for whom respondents initiated treatment followed the expected trend, according to a locally weighted scatterplot smoothing (additional file 1) and were negatively correlated (Spearman’s ρ = − 0.42, *P* < 0.001). Likewise, the percentage of dogs that respondents reported starting treatment in, and starting their own dog on insulin should it develop DM, followed the expected trend according to a locally weighted scatterplot smoothing (additional file 1) and were positively correlated (Spearman’s ρ = 0.40, *P* < 0.001). These correlations indicated a logical consistency in the respondents’ answers.

### Treatment

**Fig. 1:**
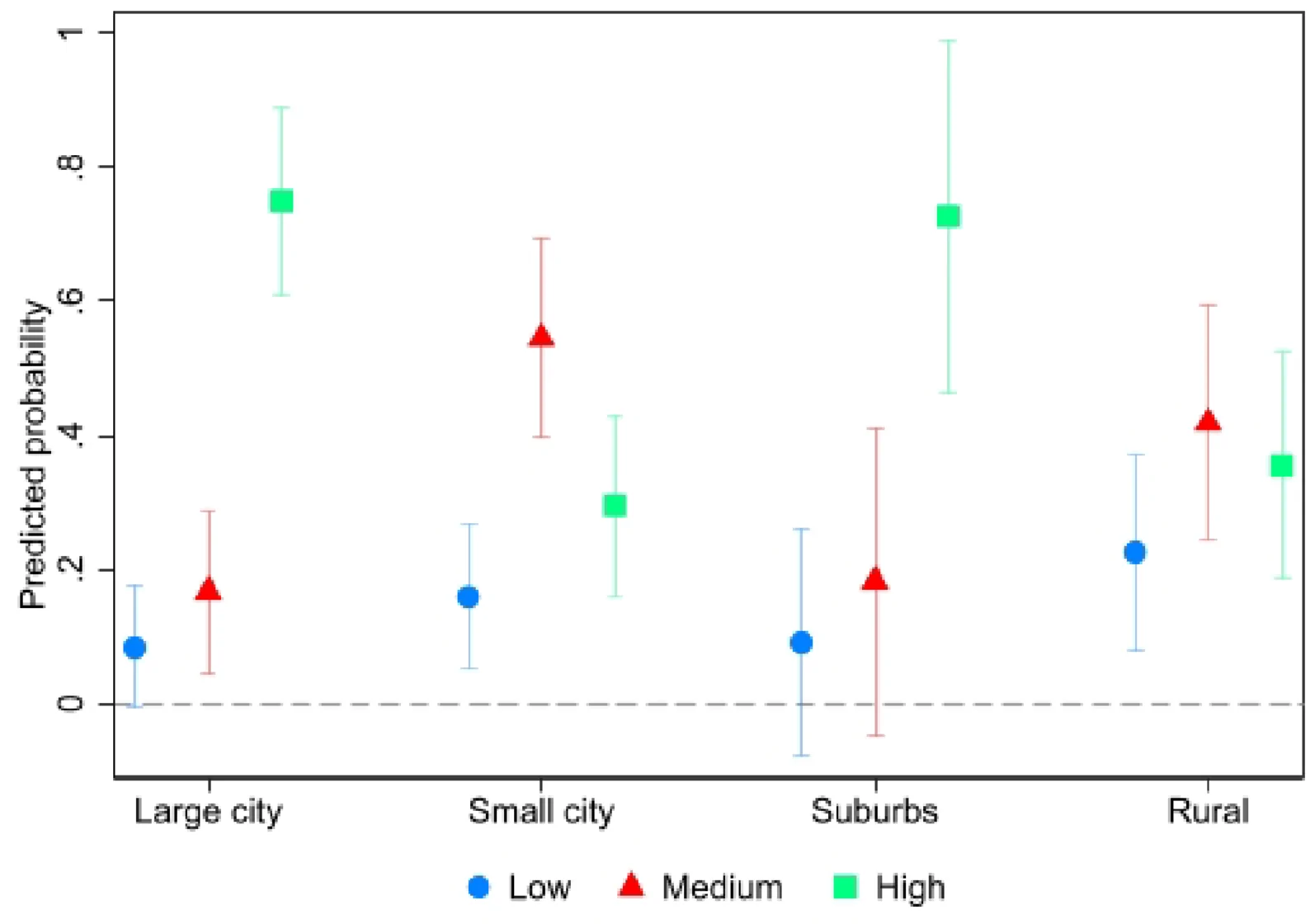
Modified margins plot showing predicted probabilities of grouped treatment initiation percentages across practice locations. The vertical lines represent the 95% confidence intervals. The circular markers denote the low group (number of respondents in the group = 18), triangles the medium group (*n* = 45) and squares the high group (*n* = 59). The low group included 0–29% treatment initiation rates, 30–89% and ≥ 90% in the medium and high groups, respectively. Large city (*n* = 36) and suburban practitioners (*n* = 11) were more likely to report high treatment initiation percentages, with rural (*n* = 31) and small city (*n* = 44) practitioners more likely reporting medium initiation percentages

Nearly half (48.8%, *n* = 61/125) of the respondents reported starting treatment in ≥ 90% of dogs diagnosed with DM, while 18.4% (*n* = 23) initiated treatment in 70–89%. Of the remaining respondents, 12% initiated treatment in 50–69% (*n* = 15), 6.4% in 30–49% (*n* = 8), and 5.6% (*n* = 7) in 10–29%. Few respondents (8.8%, *n* = 11) initiated treatment in less than 10% of DM patients. Overall, the estimated cumulative incidence risk for treatment initiation was 71.9% (95% CI 63.2, 80.6). As displayed in Fig. 1, treatment initiation percentages differed across practice locations. Respondents working in a large city or suburbs had a higher probability of reporting high frequencies of treatment initiation compared to respondents working in small cities and in rural areas. The estimated probability of treatment initiation appeared to be similar across type of practice (small animal, mixed, or on-call only; figure shown in additional file 1).

The majority of the respondents (*n* = 90, 76.2%) would treat their own dog with insulin if it developed DM (44%, *n* = 52 definitely yes, 32.2%, *n* = 38 most likely), and 22% (*n* = 26) was uncertain. Only two veterinarians stated they would definitely *not* treat their own dog with insulin.

A median of 9/10 dogs that were started on treatment were reported to be started on a new diet (*n* = 125 respondents). None were reported to start oral hypoglycemic medications (IQR 0–0, *n* = 121 respondents), whilst respondents reported starting insulin in all treated dogs (10/10, IQR 8–10; *n* = 124 respondents).

Generally, veterinarians perceived both insurance status (7/10, IQR 5–8) and whether an owner or someone in the owners’ social network had DM (7/10, IQR 4–8), to have a major impact on whether an owner would treat their dog with insulin. Furthermore, having had a dog or a cat before with DM was perceived to have moderate effect on whether to start treatment (5/10, IQR 2–8). Responses are summarised in Table 2.

**Table 2:** Perceived owner-related influences on the decision to treat and impacts on quality-of-life

Parameter	Median number (IQR)
On a scale of 1 to 10: Are owners with an insured dog more likely to start insulin therapy?	7 (5–8)
On a scale of 1 to 10: Are owners more likely to start treatment if themselves or a human in their close social circle have diabetes?	7 (4–8)
On a scale of 1 to 10: Are owners more likely to start treatment if they have or know of someone that has (had) a cat or dog with diabetes?	5 (2–8)
Of 10 dogs started on insulin, how many dogs achieve good quality of life with insulin therapy?	7 (5–8)
Of 10 owners how many owners expresses that their lifestyle is NOT affected by insulin injections?	4 (1–7)

### Neutering

When asked “out of 10 intact female dogs with diabetes, how many are neutered?”, a median of 10 dogs was reported, with an interquartile range of 8–10. Of the 46 veterinarians stating that not all female intact dogs in their practice were neutered near or at the time of diagnosis, 43 provided 95 answers as to why (multiple answers possible). The most common reasons for not neutering were cost (*n* = 29, 30%), owner unwilling for the dog to be neutered (*n* = 23, 24%), lack of understanding the importance of neutering (*n* = 19, 20%), and owner concerns about impact on the dog’s quality of life when combining insulin therapy with neutering (*n* = 17, 17.8%). Of the open-ended responses (*n* = 7, 8.2%), four veterinarians stated that the age of the dog was a limiting factor, and three reported that they started insulin therapy first, before considering neutering, to assess whether the owner was able and willing to care for a diabetic dog.

### Veterinary and perceived owner concerns regarding treatment of a diabetic dog

**Fig. 2:**
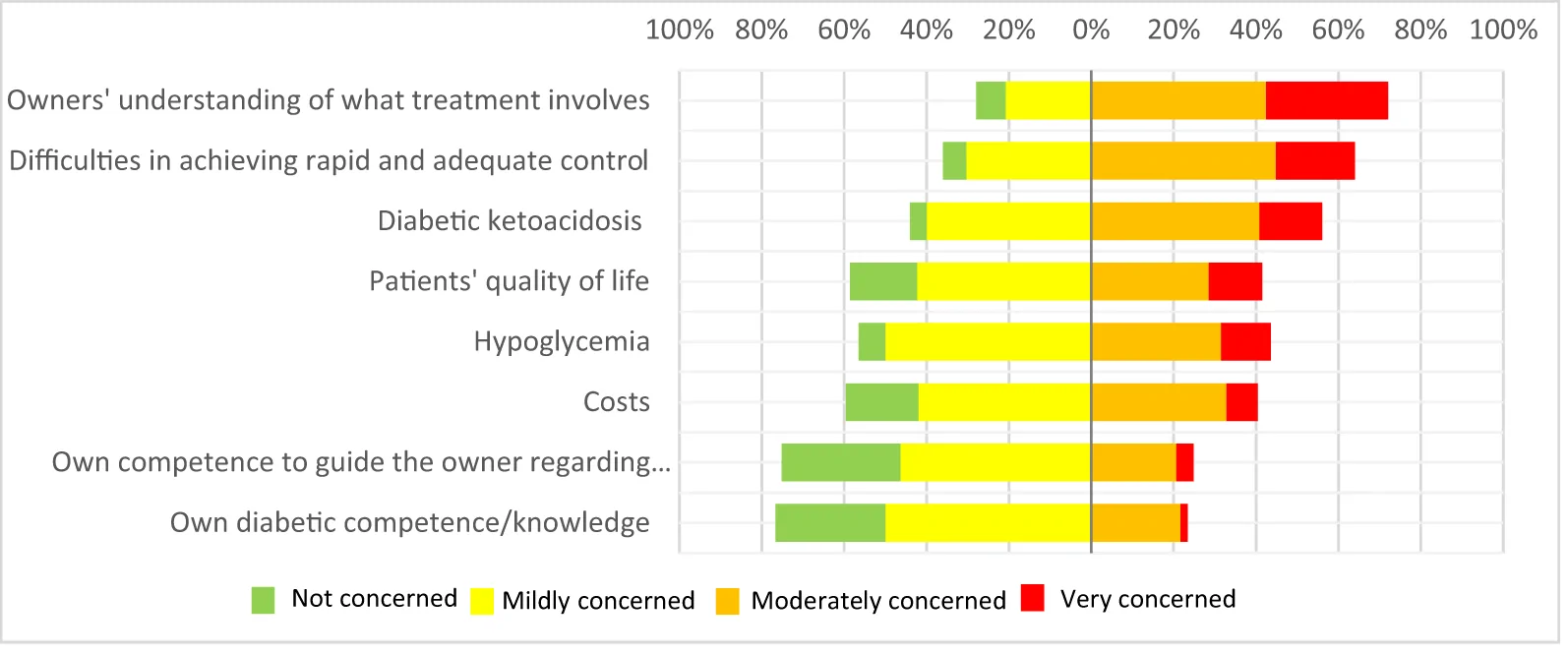
Practising veterinarians’ degree of concern regarding treatment-related issues. The respondents were asked: “To what degree are you worried about the following issues when treating a diabetic dog?”

Figure 2 displays veterinarian concerns regarding treating a dog with DM. Their main concerns were “difficulties in achieving rapid and adequate control” and “owners’ understanding of what treatment involves”.

**Fig. 3:**
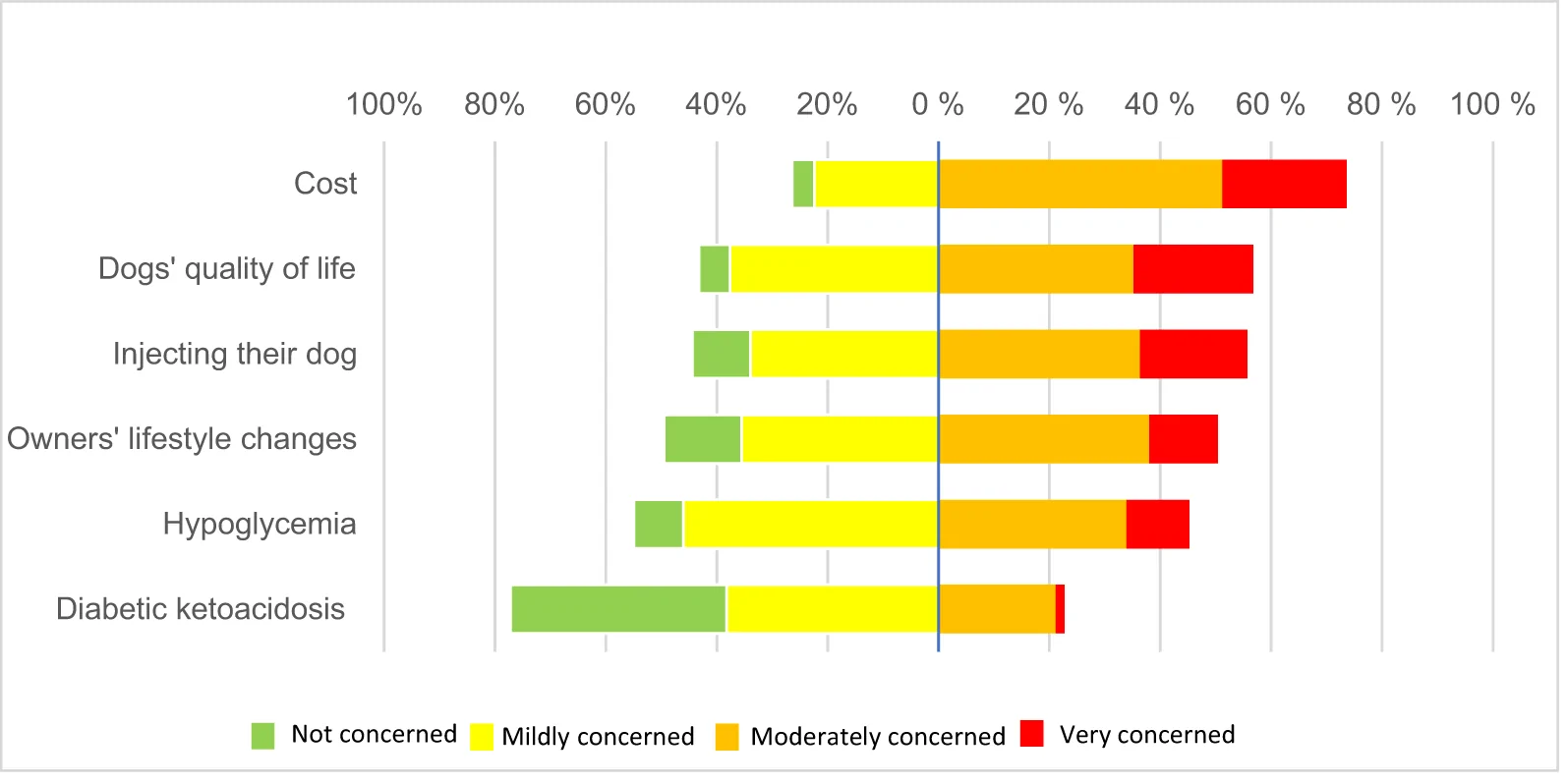
Perceived degree of owner concerns regarding diabetes-related issues. Practising veterinarians were asked “According to you or based on your experience, to what degree are owners of diabetic animals concerned by the following issues?”

Perceived main concerns for owners were cost, injecting their dog, and their dogs’ quality of life (Fig. 3).

### Monitoring

Respondents’ preferred monitoring methods (273 answers given, multiple answers possible) were at-home BGC (*n* = 73, 26.7%), CGM (*n* = 61, 22.3%), and serum fructosamine concentrations (*n* = 56, 20.5%). Less preferred methods were urine glucose (*n* = 41, 15%) and BGC at the clinic (*n* = 39, 14.3%), and three respondents did not provide an answer (1.1%). Nearly one third of the respondents (31%, *n* = 37) answered “yes” to the question “In your practice, has an owner ever had their dog euthanised due to problems with monitoring their dog?” Thirty-five respondents provided information about what type of monitoring they had used for these patients (85 answers given, multiple answers possible): The three most common forms of monitoring for these dogs were at-home BGC (*n* = 26, 30.5%), BGC at the clinic (*n* = 19, 22.4%) and serum fructosamine concentrations (*n* = 15, 17.6%).

Most respondents (*n* = 76/125, 60.8%) had never used CGM, and for 43.4% (*n* = 33) the reason was this being new and unfamiliar equipment, or a technique they were not comfortable with. Just over one quarter (26.3%, *n* = 20) provided an open-ended reply for not using CGM, with the majority describing that the owners didn’t want to have a sensor placed on their dog. Other reasons commonly reported were cost (10.5%, *n* = 8) or difficulties obtaining a sensor (10.5%, *n* = 8). Finally, 9.2% (*n* = 7) had never heard of CGM. Of the veterinarians that had used CGM (*n* = 49), 87.5% (*n* = 43) had somewhat positive to positive experiences. Table 3 shows the distribution of positive and negative CGM experiences (multiple answers possible).

**Table 3:** Practising veterinarians’ experience with use of CGM

Positive experiences with CGM	Of total respondents (*n* = 48)
Dog achieves better quality of life by avoiding needle sticks	66.7% (*n* = 33)
More data is available for the assessment of treatment response	85.4% (*n* = 41)
Owner seems less worried about complications like hypoglycaemia	68.8% (*n* = 33)
Increases the communication between you as a vet and the owner, which again improves compliance	56.3% (*n* = 27)

Negative experiences with CGM	Of total respondents (*n* = 46)
Increased costs	47.8% (*n* = 22)
Owner gets hung-up on results and requires very close follow-up	52.2% (*n* = 24)
Owner gets hung up on results and changes the insulin treatment themselves	50.0% (*n* = 23)
Owner seems more worried about complications, e.g. because of visualisation of changes in glucose	37.0% (*n* = 17)
Sensor functionality (e.g. stops working or falls off) is too short	63.0% (*n* = 29)

The most common reasons for a CGM to be replaced before it had run out (multiple answers possible) was “the sensor falls off before 14 days has passed” (65.6%, *n* = 40/61), “the sensor stopped working” (24.5%, *n* = 15/61), and “the sensor showed inaccurate measurements” (6,5%, *n* = 4/61). Most respondents (91%, *n* = 41/45 respondents) changed the CGM sensor at the clinic, either themselves or had it done by a veterinary nurse or assistant. One of the respondents sent the dog to another veterinary hospital for it to be replaced, and one had taught the owners how to change the sensor themselves. 80% (*n* = 35/44) used tissue glue when attaching the sensor. Most (72.4% *n* = 34/47) respondents stated that they did not perceive any pain or discomfort upon sensor placement. 63% of respondents had never, or in less than 10% of patients, noted skin reactions after placement (*n* = 28/44), while one respondent (2.3%) noted it in 70–90% of the patients. 94% of respondents (94.4%, *n* = 118/125) would be positive to the use of a subcutaneous glucose sensor if it became more readily available, the remainder stated they were uncertain (5.6%, *n* = 7). Perceived cost of CGM per month accepted by owners was less than 500 NOK (approximately 43 Euro) for 25% of the respondents (*n* = 31/124), between 500 and 1,499 NOK (43 and 130 Euro) for 58% (*n* = 72/124) and 12% (*n* = 15) thought owners would accept more than 1.500 NOK (> 130 Euro) per month. Six respondents provided free-text responses: five stated that the answer was dependent on the level of insurance coverage, and one stated they did not know.

### Prognosis

When asked about their opinion on the prognosis for dogs with DM in general, 120 responded, with the majority stating guarded to good (*n* = 68, 56.67%). Similar percentages considered the prognosis good (*n* = 23, 19.17%) and guarded (*n* = 21, 17.5%), while seven (5.83%) and one (0.8%) considered it guarded-to-poor and poor, respectively. When asked whether they believed female entire dogs to have a better prognosis, 38.7% (*n* = 45) responded no, 28.4% (*n* = 33) yes, and 32.7% did not know (*n* = 38).

### Euthanasia

For dogs started on insulin therapy, the estimated cumulative risk of euthanasia within a month was 11.5% (95% CI 8.2, 14.7); and the estimated cumulative euthanasia risk within a year was 26.9% (95% CI 22.0, 31.9). Most of the dogs were euthanised due to owners’ wishes (90%, IQR 70–100%); 30% (IQR 10–60%) were due to veterinary recommendation (given as two separate questions).

**Fig. 4:**
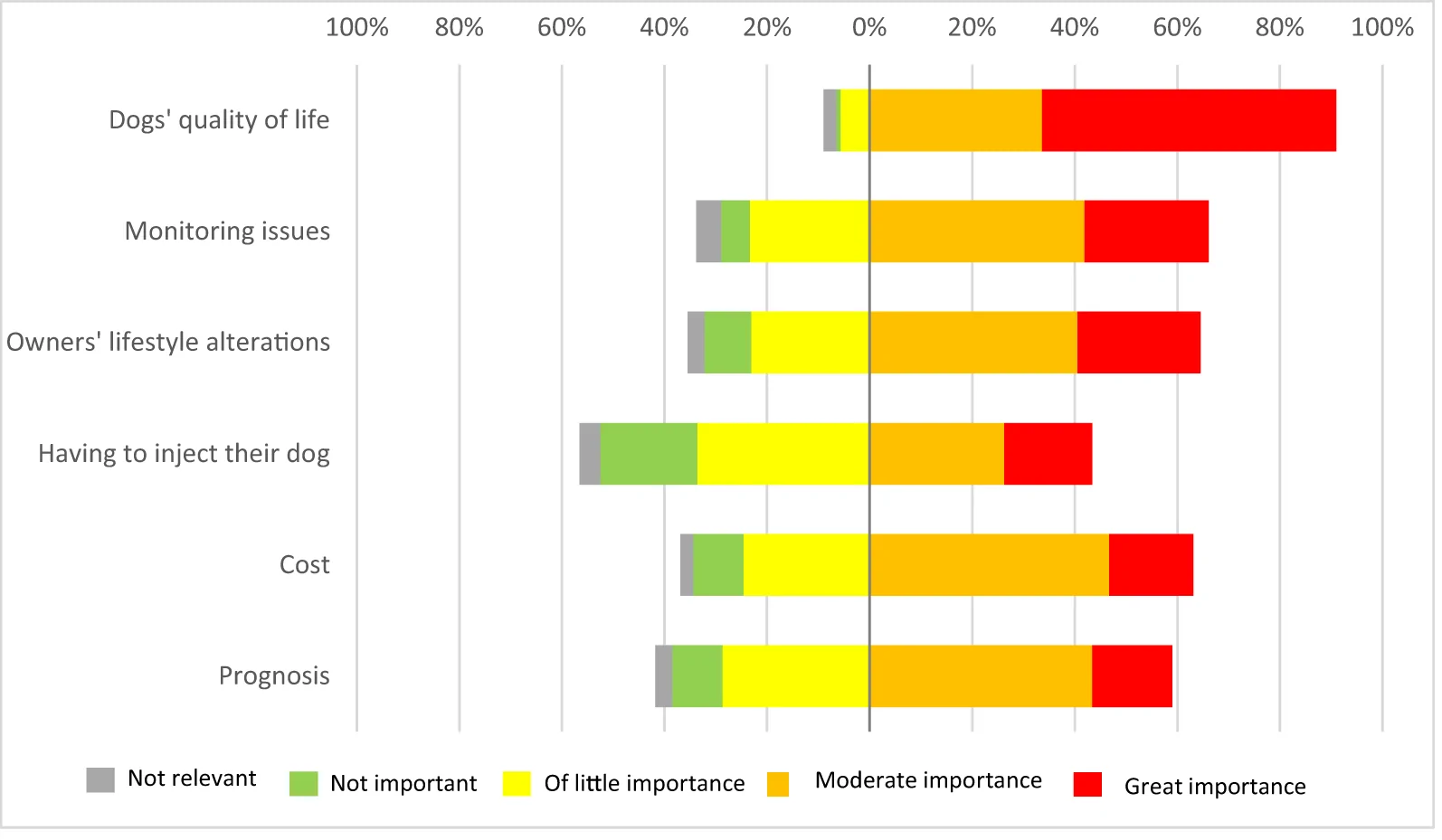
Practising veterinarians’ perceived main factors influencing owners’ decision to euthanise their diabetic dog

**Fig. 5:**
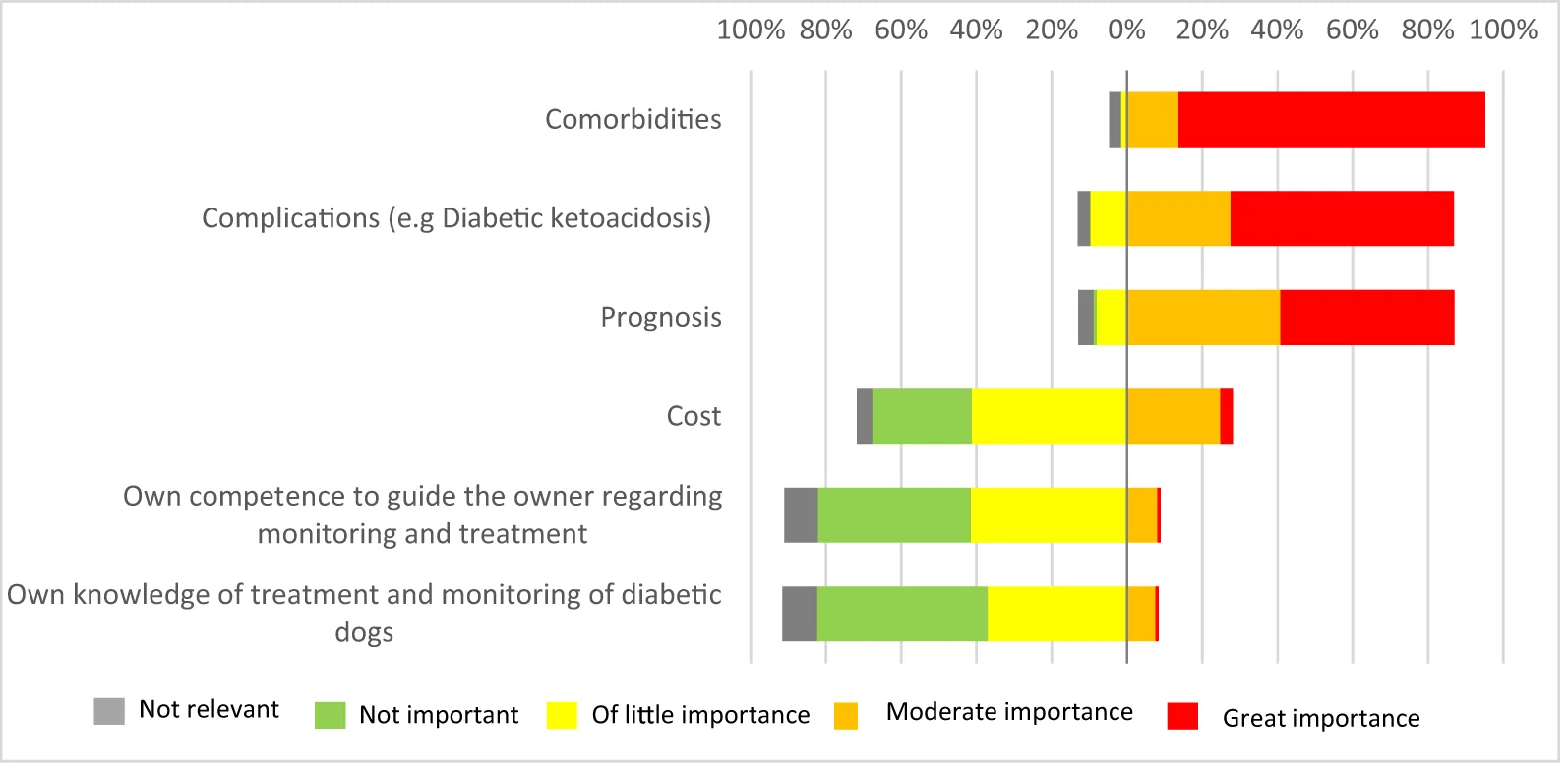
Main reported factors influencing veterinary decision to recommend euthanasia

Perceived main owner triggers for euthanasia were dogs’ quality of life (91%, *n* = 111/122, rating this of moderate or great importance), monitoring (66.1%, *n* = 82/124, rating this of moderate or great importance) and owners’ lifestyle changes (64.4%, *n* = 111/122, rating this of moderate or great importance, Fig. 4). For veterinarians, comorbidities were the leading factor (81.5%, *n* = 101/124 rating this of great importance) for recommending euthanasia (Fig. 5).

**Fig. 6:**
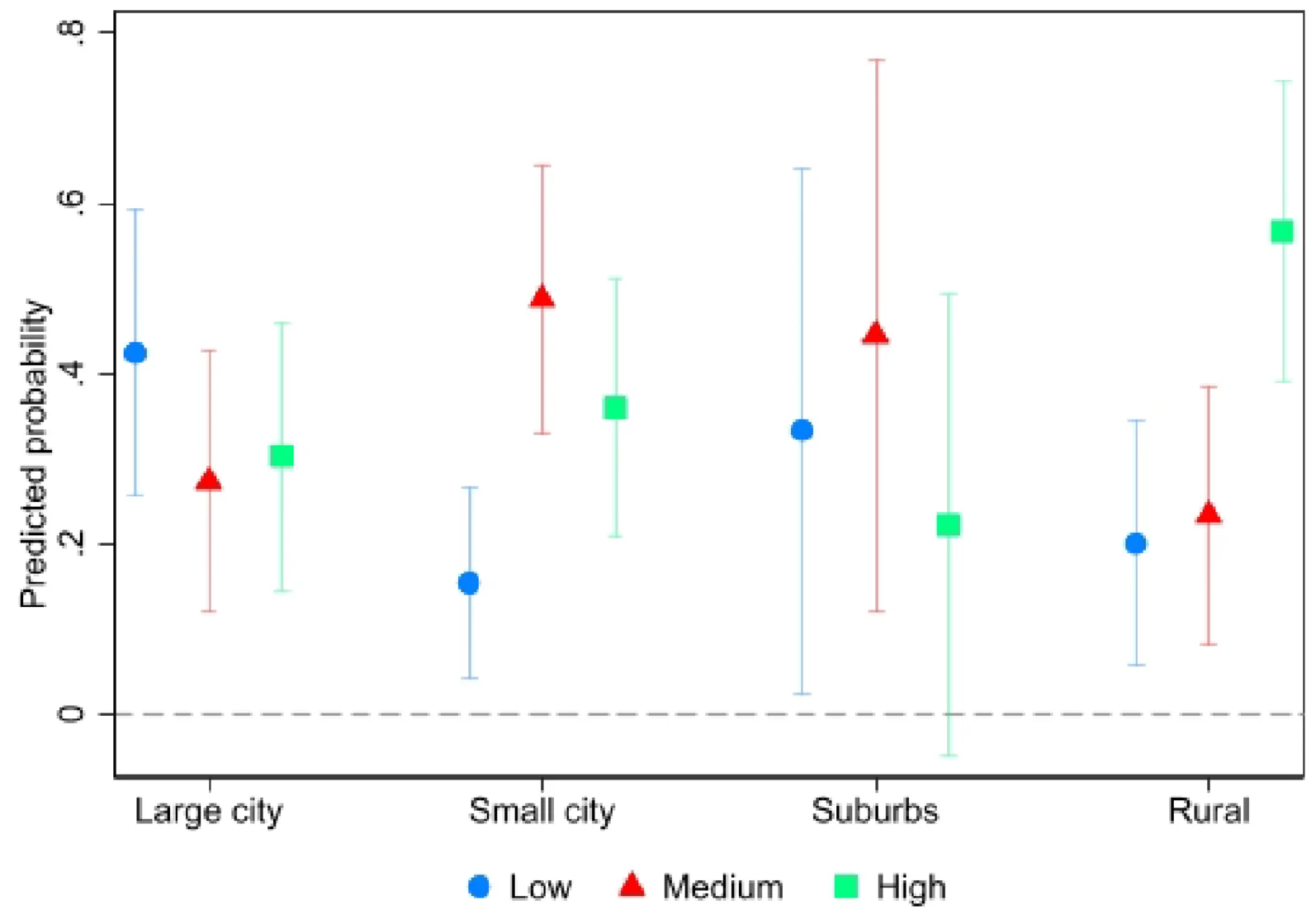
Modified margins plot showing predicted probabilities of grouped euthanasia percentages across practice locations. The vertical lines represent the 95% confidence intervals for the predicted mean. The circular markers denote the low probability group (number of respondents in the group = 29), triangles the medium probability group (*n* = 39) and squares the high probability group (*n* = 43). The low group included 0–30% euthanasia percentages, 40–60% and 70–100% in the medium and high groups, respectively. Respondents working in rural areas (*n* = 30) were more likely to report high euthanasia percentages, small city (*n* = 39) and suburban respondents (*n* = 9) were more likely to report medium percentages and large city practitioners (*n* = 33) were more likely to report low percentages

The relationship between reported predicted probability of euthanasia percentages and practice locations is visualised in Fig. 6. The predicted probabilities indicate that respondents working in rural areas had a higher likelihood of reporting higher percentages of euthanasia, small city respondents had a higher likelihood of reporting medium percentages and respondents working in a large city had higher probability of reporting low euthanasia percentages. Predicted euthanasia probabilities appeared not to differ across types of practice (figure shown in additional file 1).

## Discussion

This questionnaire-based study aimed to estimate the frequency of euthanasia of dogs due to DM, describe Norwegian veterinarians’ perceptions regarding treatment and monitoring habits, and describe how treatment and euthanasia vary with demographics. Our results showed that, based on the respondents’ perceptions, approximately 70% of dogs with DM are started on treatment. This suggests that a significant proportion of dogs are not started on treatment and therefore are likely to be euthanised around the time of diagnosis. The proportion of dogs euthanised is not necessarily the reciprocal of those started on treatment; the omitted ‘out of 10 newly diagnosed dogs with DM how many are euthanised at the time of diagnosis?’ would have directly addressed this question. Nevertheless, there are no viable alternatives to insulin treatment in diabetic dogs, and therefore very likely that euthanasia is performed when treatment is not initiated. Naturally, the dog being taken to a different veterinarian (than the respondent) and started on insulin could occur. Thus, our estimate is associated with some uncertainty. This estimate of the (presumable) risk of euthanasia at the time of DM diagnosis falls in between the around 40% reported in 2007 among insured Swedish dogs [13], and the 10% estimated by the Big Pet Diabetes Survey, 2017 [6]. Furthermore, we found that the cumulative risk of euthanasia within the first year of DM-diagnosis, after starting insulin treatment, was approximately 27%, suggesting a euthanasia percentage within the first year of all diabetic dogs (treated and euthanised at the time of diagnosis) close to 50% in our study population. In comparison, a median of 2 of 10 dogs (1 of 10 at diagnosis and 1 of 10 within the first year) were estimated to be euthanised in the aforementioned study [6]. This difference is substantial and should not only be ascribed to the bias. This difference is possibly explained by the fact that the majority of respondents in the study from Niessen et al. [6] were from the USA, UK, Ireland and Canada; likely reflecting demographic and geographic, cultural and political differences, as well as different ethical views when it comes to treatment of animals. Sweden and Norway are geographical neighbours and share many of these views, which may explain the similarity in euthanasia risks observed in these countries. The somewhat lower percentage euthanised at the time of diagnosis in our population compared to that reported in Sweden in 2007 [13], could reflect biased estimates or random error, changes in political, cultural and ethical values, but also development and availability of treatment strategies and monitoring options over the last (nearly) 20 years.

As reported in the Big Pet Diabetes Survey [6], we also found differences within our study population between rural and urban locations of the veterinary practice, with lower probability of treatment initiation and higher probability of euthanasia in rural areas and small cities. This may reflect differences in the clients’ purpose of keeping a dog (e.g. working dog vs. companion dog), expectations, attitudes, economy, and practice differences [14, 15]. The majority of veterinarians responding to our study reported diagnosing less than three dogs with DM per year. This low number of cases is likely to affect the study’s generalisability; a low number of cases seen by a veterinarian will have a greater relative effect on the given response than by practitioners seeing a larger number of cases, as the questionnaire relies on each veterinary practitioner’s recollected experience. Consequently, limited exposure to DM cases is likely to affect reliability and validity of the responses, especially with respect to treatment and euthanasia risks. The estimated treatment initiation probability, one month and one year euthanasia risks are therefore likely more representative as it takes into account the number of cases seen per year. Furthermore, a low number of cases seen per year could affect the practitioner’s confidence in treating diabetic dogs. Of the respondents, 46.3% and 50% reported that they were mildly, 20.7 and 21.7% moderately, and 4.1% and 1.7% markedly concerned about their own ability to guide owners regarding therapy, and their own diabetic competence, respectively. This uncertainty highlights the importance of post-graduate education, as well as advisory support from specialists to general practitioners, regarding monitoring and treatment options. The intensity of different protocols is also likely to affect euthanasia rates. Therefore, the opportunity to discuss optimal treatment and monitoring protocols for each diabetic pet and owner individually is of great value: there will be individual preferences regarding costs, injections, lifestyle, and other considerations differing between owners [5, 6].

We found that owners having had a diabetic pet previously, or with diabetic people among their close family or friends, were perceived to be more likely to treat their dog with insulin. It should be noted that this is a reported effect and veterinarians are likely not to measure or recall this information accurately. Before concluding that this effect is real, further research measure the presence of these features among owners and link them to their decisions regarding treatment of their diabetic dog.

Intact female dogs with DM clearly benefit from neutering due to the anti-insulin effects of progesterone and (mammary) growth hormone [16]. Neutering improves diabetic control and has the potential to induce diabetic remission [16–18], and it has been shown that neutering closer to the time of diagnosis may increase the likelihood of remission [16]. Routine neutering is prohibited by law in Norway (i.e., a medical reason is needed). In our study, nearly all veterinarians reported neutering all intact female dogs diagnosed with and treated for DM. Veterinarians reporting that they did *not* neuter all dogs, stated costs and owners’ unwillingness for the dog to undergo neutering as the most common reasons. As the possibility of remission tends to be a welcome hope for owners of newly diabetic animals, it would seem prudent to include these aspects when discussing neutering of a female intact diabetic dog with the owner. Finally, the high percentage of perceived unwillingness for a dog to undergo neutering might also have a cultural aspect; it may be that the neutering prohibition makes Norwegian dog owners more worried or negative towards this procedure.

We found that nearly one third of the respondents answered “yes” to the question “In your practice, has an owner ever had their dog euthanised due to problems with monitoring their dog?”. Blood glucose curves measured at home or in the clinic were the most common forms of monitoring by the practitioners that responded yes to the previously mentioned question. A previous study [10] showed that owners perceived CGM to be less stressful for their pets compared to blood glucose curves, with the present study finding similar opinions among practitioners in Norway. This aspect could also be important for the overall experience for owners of diabetic dogs and may potentially prevent euthanasia. The question could, on the other hand, have been interpreted more broadly by some respondents, including problems related to diabetic control or consequences of the monitoring, leading to an overestimation of positive replies. Regardless, in dealing with both monitoring and diabetic control, it is important to remember the goals of treatment: normalisation of clinical signs and achievement of a good quality of life. These goals can, in many dogs, be achieved without intense monitoring and highlights the need for information for both practitioners and owners on different monitoring options and protocols. Of the more challenging aspects of CGM use, increased cost was perceived to be a negative factor by about half of our respondents, which is higher than what was reported by owners of diabetic dogs in Italy (36%) [10]. Approximately 50% of veterinarians having used CGM also reported that owners became obsessed with results, and either changed the insulin treatment themselves or needed close follow-up by the veterinary practitioner. Similar findings have been reported by others [19], with 43.7% of respondents reporting difficulties in managing owners’ anxiety. The fact that as many as half of diabetic dog owners changing insulin doses themselves, highlights another important challenge for veterinarians. Uneducated insulin dose adjustments can potentially be life-threatening, and measures should be taken to educate owners about this prior to CGM-placement. Finally, early detachment or sensor malfunction has commonly been reported, as was also seen in our study [10, 20]. Placement of a subcutaneous sensor (e.g. Eversense^®^ XL) negates this problem, and near all veterinarians were positive to a subcutaneous sensor should it be readily available for the use in dogs.

Although the responses to our questionnaire highlights issues with treatment and monitoring that may be adjusted to improve the care for dogs with DM, it is important to note that our study has several limitations. Only a limited number of questions regarding demographics were included in this questionnaire and the representativeness of the sampled population in comparison with the target population is therefore uncertain. However, we obtained a distribution of small animal (70.8%) vs. mixed practice practitioners (19.3%) that was similar to a recent questionnaire-based study of Norwegian veterinarians’ suicidal thoughts and attitudes [21]. This study reported a response rate of 75%, with 802 and 268 of responding veterinarians working in companion animal and mixed clinical practice, respectively. Our response rate was low, however, if we compare the response rate in this study to other online distributed canine DM-related questionnaires, we find a similar, or likely even lower response rates in these studies [6, 19]. Explanations for low response rates may include lack of effective marketing, and that the questionnaire was online submission only.

The low response rate in our study is likely to have influenced our results through non-response bias or self-selection. The representativeness of our sample must be considered, as it is likely that veterinarians with stronger opinions or interest in canine DM more likely to respond. As such, veterinarians more engaged or involved in cases with DM than the average population are more likely to be represented. This has probably influenced, in particular, our estimated treatment initiation and euthanasia risks, which is likely somewhat over- and underestimated, respectively. This will limit the representativeness of our study. Furthermore, recall bias must be considered; occurring when participants do not accurately remember a past event or experience. As about 15% of participants in this study stated they would only diagnose canine DM once every two years, details regarding treatment concerns or reasons for euthanasia could be inaccurate. Also, a number of our questions asked about the veterinarian’s perception of an owner’s attitude and decision making, which might not be accurate.

Our study used (mostly) closed questions, which may have biased the results by potentially restricting the respondents’ responses. All questions had an option of “other” for the participants to write out additional information or comments, which could avoid some of the potential biases from the closed format. Assessing these “other” responses, however, no consistent misunderstandings or omissions were identified, except regarding one question: “Often euthanasia is a combination of the factors given in the previous questions, but of these, what is the most important factor for you?”. The response “comorbidities” was by mistake omitted from the final online form, and therefore many respondents wrote “comorbidities”, or stated that this was missing. As described, this question was excluded from the final analysis.

## Conclusions

In conclusion, this questionnaire-based study describes perceived treatment concerns, and reasons for and estimates of euthanasia risks in dogs with DM, based on the experiences of veterinary practitioners working in Norway. Although a questionnaire has several limitations, the results highlight important aspects of the treatment and monitoring of dogs with DM, as well as triggers for euthanasia. Further investigations into the aspects identified could be useful to improve the overall care for diabetic dogs.

## Supplementary Information

Below is the link to the electronic supplementary material.

Supplementary Material 1.

Supplementary Material 2.

## Data Availability

The datasets used and analysed during the current study are available from the corresponding author on reasonable request.

## References

[1] Heeley AM, O’Neill DG, Davison LJ, Church DB, Corless EK, Brodbelt DC. Diabetes mellitus in dogs attending UK primary-care practices: frequency, risk factors and survival. Canine Med Genet. 2020;7:6.

[2] Mattin M, O’Neill D, Church D, McGreevy PD, Thomson PC, Brodbelt DC. An epidemiological study of diabetes mellitus in dogs attending first opinion practice in the UK. Vet Rec. 2014;174:349. 24570406 10.1136/vr.101950

[3] Catchpole B, Ristic JM, Fleeman LM, Davison LJ. Canine diabetes mellitus: can old dogs teach us new tricks? Diabetologia. 2005;48:1948–56. 16151773 10.1007/s00125-005-1921-1

[4] Yoon S, Fleeman LM, Wilson BJ, Mansfield CS, McGreevy P. Epidemiological study of dogs with diabetes mellitus attending primary care veterinary clinics in Australia. Vet Rec. 2020;187:e22. 32051292 10.1136/vr.105467

[5] Niessen SJ, Powney S, Guitian J, Niessen AP, Pion PD, Shaw JA, et al. Evaluation of a quality-of-life tool for dogs with diabetes mellitus. J Vet Intern Med. 2012;26:953–61. 22646241 10.1111/j.1939-1676.2012.00947.x

[6] Niessen SJM, Hazuchova K, Powney SL, Guitian J, Niessen APM, Pion PD, et al. The Big Pet Diabetes Survey: perceived frequency and triggers for euthanasia. Vet Sci. 2017;4:27. 29056686 10.3390/vetsci4020027 PMC5606606

[7] Del Baldo F, Canton C, Testa S, Swales H, Drudi I, Golinelli S, et al. Comparison between a flash glucose monitoring system and a portable blood glucose meter for monitoring dogs with diabetes mellitus. J Vet Intern Med. 2020;34:2296–305. 33124730 10.1111/jvim.15930 PMC7694810

[8] Corradini S, Pilosio B, Dondi F, Linari G, Testa S, Brugnoli F, et al. Accuracy of a flash glucose monitoring system in diabetic dogs. J Vet Intern Med. 2016;30:983–8. 27318663 10.1111/jvim.14355 PMC5094557

[9] Del Baldo F, Fracassi F. Continuous glucose monitoring in dogs and cats: application of new technology to an old problem. Vet Clin North Am Small Anim Pract. 2023;53:591–613. 36854635 10.1016/j.cvsm.2023.01.008

[10] Re M, Del Baldo F, Tardo AM, Fracassi F. Monitoring of diabetes mellitus using the flash glucose monitoring system: The owners’ point of view. Vet Sci. 2023;10:203. 36977242 10.3390/vetsci10030203 PMC10052096

[11] Otani Y, Kanamori M, Kato H, Dwyer CM. Cross-cultural variation in understanding of animal welfare principles and animal management practices among veterinary and animal welfare professionals in the UK and Japan. Anim Welf. 2025;34:e55. 40988869 10.1017/awf.2025.10026 PMC12451389

[12] Fraser D. The role of the veterinarian in animal welfare. Animal welfare: too much or too little? Abstracts of the 21st Symposium of the Nordic Committee for Veterinary Scientific Cooperation (NKVet). Vaerløse, Denmark. September 24–25, 2007. Acta Vet Scand. 2008; 50(Suppl 1): S1-12. 10.1186/1751-0147-50-S1-S1 PMC4235121 19049678

[13] Fall T, Hamlin HH, Hedhammar A, Kämpe O, Egenvall A. Diabetes mellitus in a population of 180,000 insured dogs: incidence, survival, and breed distribution. J Vet Intern Med. 2007;21:1209–16. 18196728 10.1892/07-021.1

[14] Ortega-Pacheco A, Rodriguez-Buenfil JC, Bolio-Gonzalez ME, Sauri-Arceo CH, Jiménez-Coello M, Forsberg CL. A survey of dog populations in urban and rural areas of Yucatan. Mexico Anthrozoös. 2007;20:261–74.

[15] Gorodetsky E. Epidemiology of dog and cat euthanasia across Canadian prairie provinces. Can Vet J. 1997;38:649. 9332753 PMC1576864

[16] Fall T, Hedhammar A, Wallberg A, Fall N, Ahlgren KM, Hamlin HH, et al. Diabetes mellitus in elkhounds is associated with diestrus and pregnancy. J Vet Intern Med. 2010;24:1322–8. 21054539 10.1111/j.1939-1676.2010.0630.x

[17] Pöppl AG, Mottin TS, González FH. Diabetes mellitus remission after resolution of inflammatory and progesterone-related conditions in bitches. Res Vet Sci. 2013;94:471–3. 23164637 10.1016/j.rvsc.2012.10.008

[18] Fall T, Johansson Kreuger S, Juberget A, Bergström A, Hedhammar A. Gestational diabetes mellitus in 13 dogs. J Vet Intern Med. 2008;22:1296–300. 18976285 10.1111/j.1939-1676.2008.0199.x

[19] Didier M, Re M, Fracassi F, Tardo AM, Scudder C, Niessen SJ, et al. ESVE-O-1: experiences with flash glucose monitoring system in dogs and cats: a global survey of veterinary practitioners [abstract]. J Vet Intern Med. 2024;38:3537–94.39535393

[20] Campbell C, Shoelson A, Mahony O. Complications associated with a flash glucose monitoring system in diabetic dogs. Can J Vet Res. 2023;87:260–4. 37790268 PMC10542953

[21] Dalum HS, Tyssen R, Moum T, Thoresen M, Hem E. Euthanasia of animals – association with veterinarians’ suicidal thoughts and attitudes towards assisted dying in humans: a nationwide cross-sectional survey (the NORVET study). BMC Psychiatry. 2024;24:2. 38166727 10.1186/s12888-023-05402-7 PMC10763301

