# Supplementary material for: Treatment, monitoring, and euthanasia in canine diabetes mellitus - a questionnaire-based survey among veterinarians in Norway
Source: Acta Vet Scand. 2026 Aug 31;68:35. doi: 10.1186/s13028-026-00878-1 (PMC13587334; doi:10.1186/s13028-026-00878-1)
Supplement: Supplementary file 1 [file 13028_2026_878_MOESM1_ESM.pdf]

Additional file 1

Figure 1

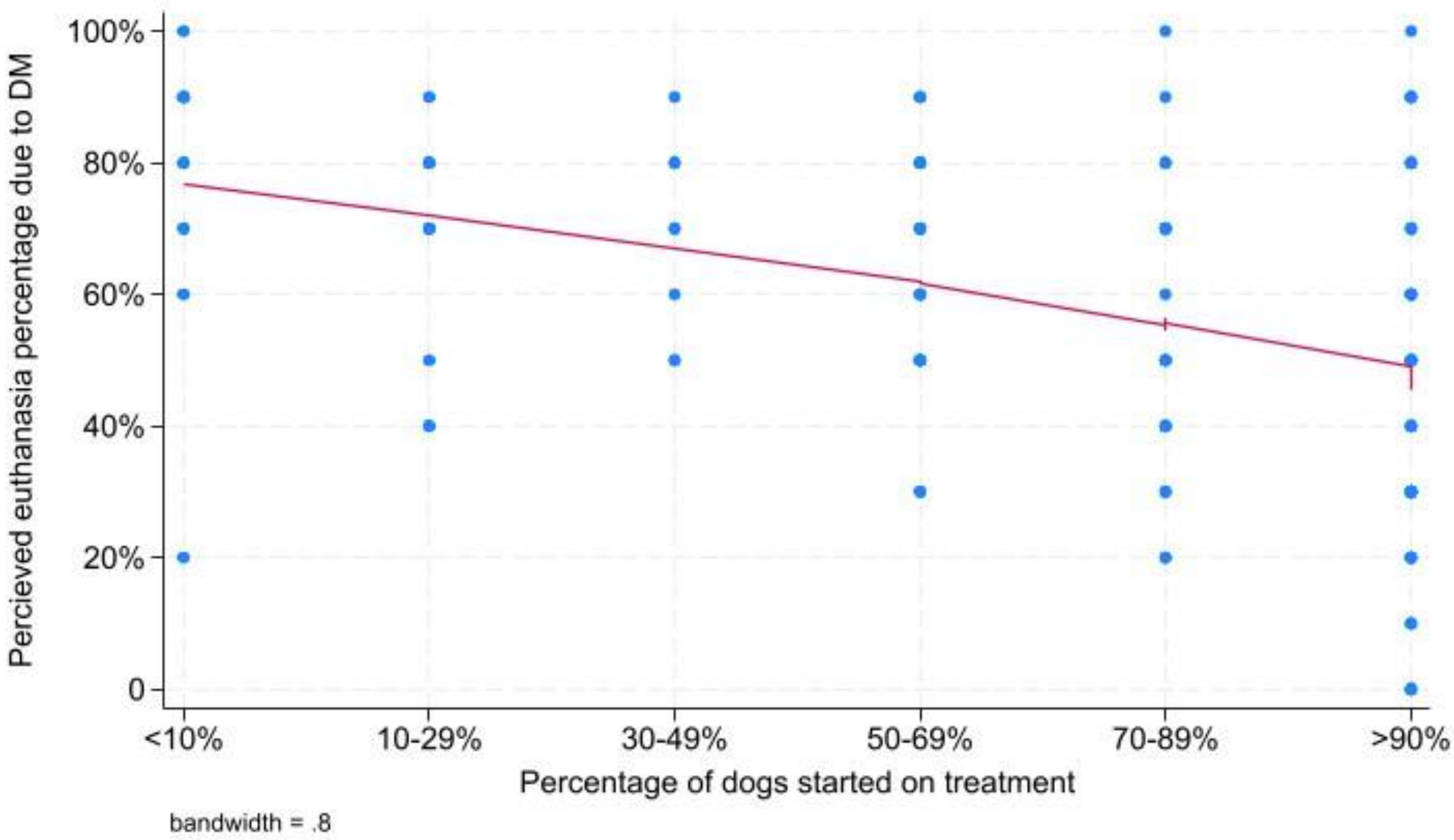

Figure 1 shows locally weighted scatterplot smoothing, bandwidth 0.8 from the questions percentage of dogs started on treatment vs perceived euthanasia percentage due to diabetes mellitus (DM)

Figure 2

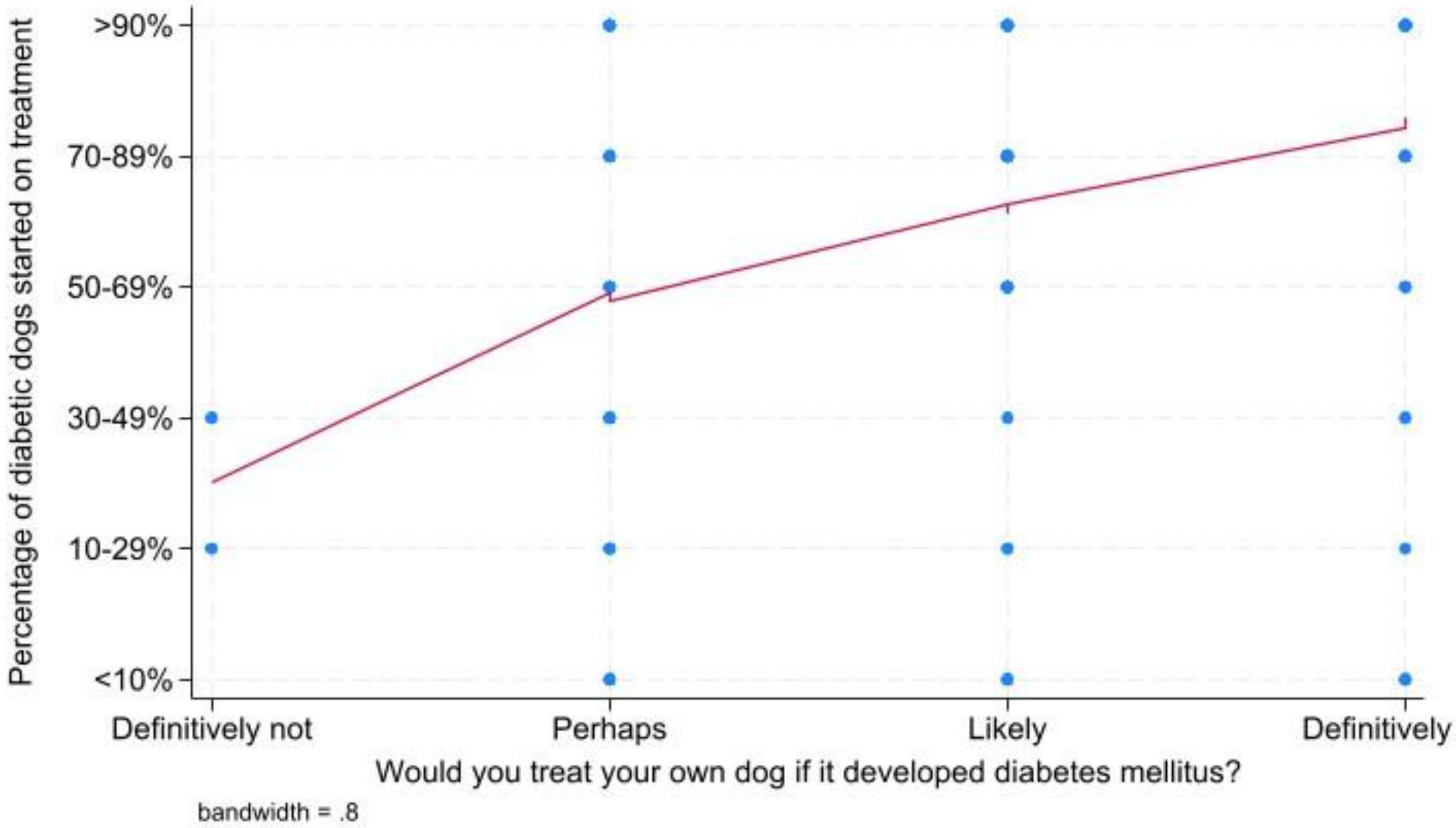

Figure 2 shows locally weighted scatterplot smoothing, bandwidth 0.8 from the questions percentage of dogs started on treatment vs perceived euthanasia percentage due to diabetes mellitus (DM)

Figure 3

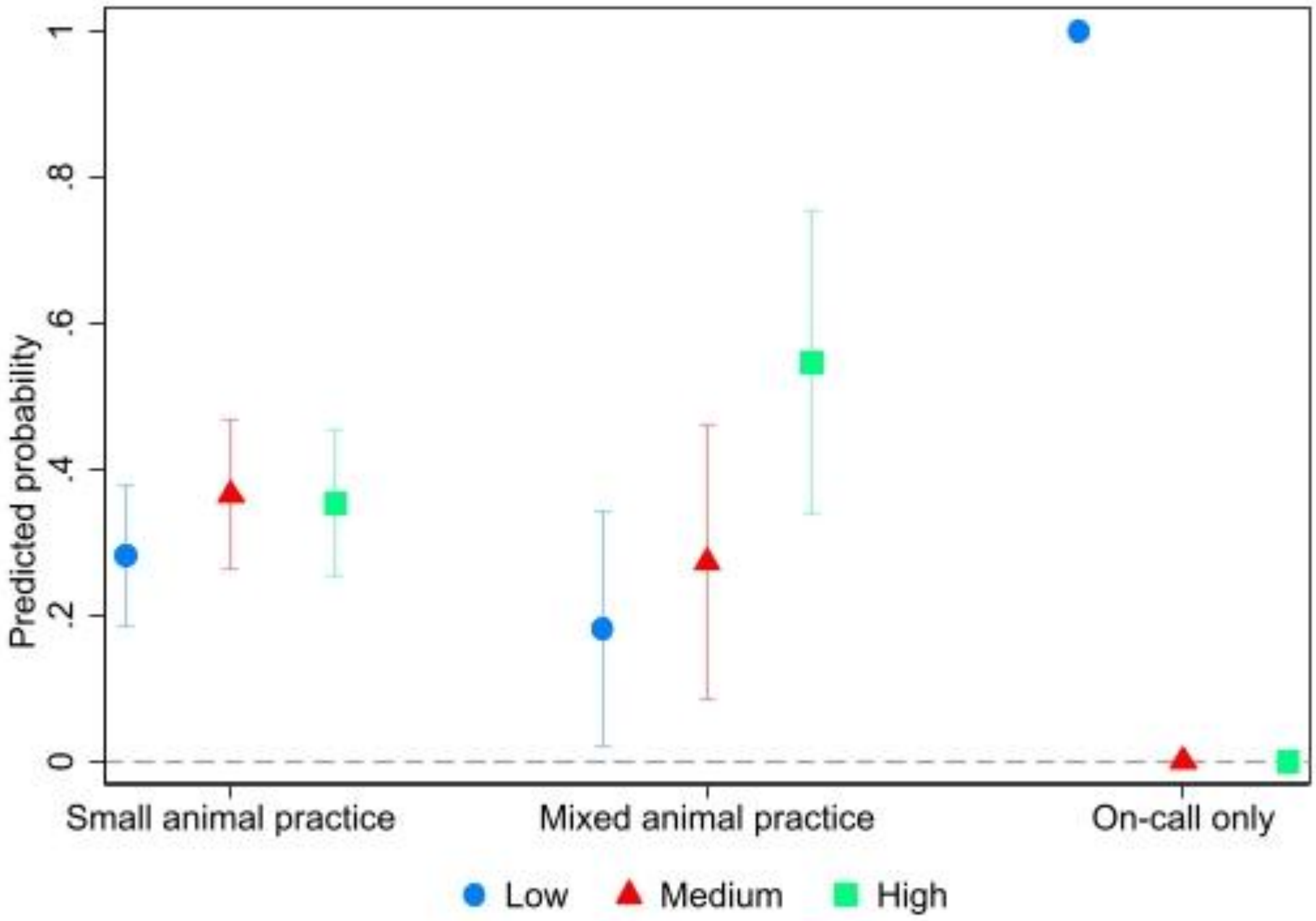

Figure 3 shows the predicted mean probabilities of grouped treatment initiation percentages against practice type. Note that "On-call only" consisted of one respondent. The vertical lines represent the 95% confidence intervals for the predicted mean. The low group included 0-29% treatment initiation rates, 30-89% and ≥ 90% in the medium and high groups, respectively.

Figure 4

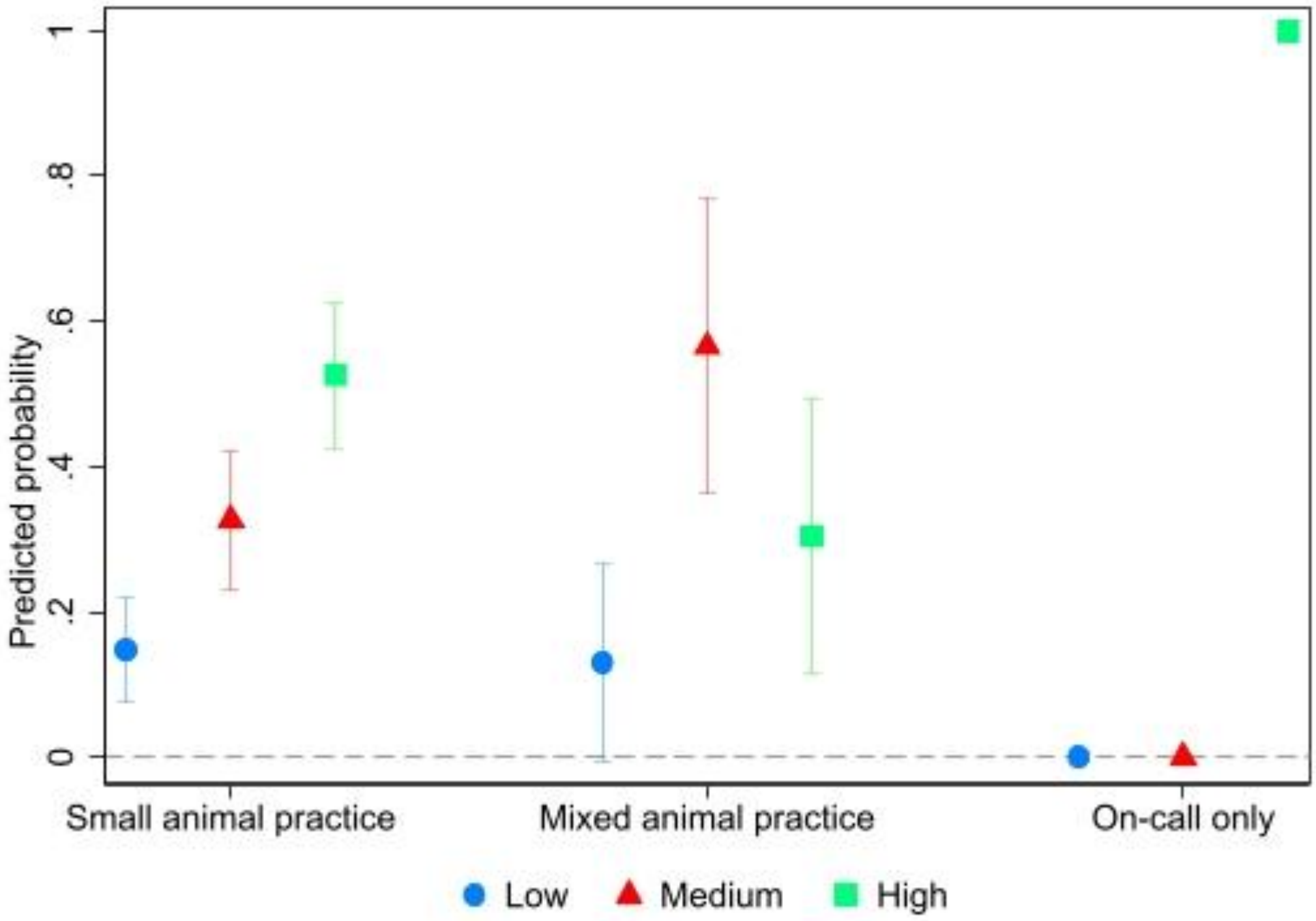

Figure 4 shows predicted mean probabilities of grouped euthanasia percentages against practice type. Note that “On-call only” consisted of one respondent. The vertical lines represent the 95% confidence intervals for the predicted mean. The low group included 0-30% euthanasia rates, 40-60% and 70-100% in the medium and high groups, respectively.
