# Supplementary material for: Treatment, monitoring, and euthanasia in canine diabetes mellitus - a questionnaire-based survey among veterinarians in Norway
Source: Acta Vet Scand. 2026 Aug 31;68:35. doi: 10.1186/s13028-026-00878-1 (PMC13587334; doi:10.1186/s13028-026-00878-1)
Supplement: Supplementary file 2 [file 13028_2026_878_MOESM2_ESM.pdf]

Treatment, monitoring, and euthanasia in canine diabetes mellitus - a questionnaire-based survey among veterinarians in Norway

Final questionnaire

Inclusion

1. **Have you ever diagnosed a dog with diabetes mellitus in your practice?** (*Har du noen gang diagnostisert en hund med diabetes mellitus?*)

a. Yes
b. No

Treatment

1. In your estimation, how many dogs with diabetes mellitus do you diagnose or start treatment per year? *(Anslagsvis, hvor mange hunder med diabetes diagnostiserer du eller starter opp behandling på per år?)*

a. < 1 dog per year (please specify under «other» if you e.g. diagnose 1 every other year).
b. 1-3
c. 3-5
d. 5-10
e. > 10
f. Other

2. Of the dogs you diagnose, in what percentage do you start any form of treatment (e.g insulin, neutering, diet)? *(Av hundene du diagnostiserer; ca. hvor stor andel utfører du en form for behandling på (for eksempel diett, kastrasjon, insulin)?*

a. None
b. < 10%
c. 10-29 %
d. 30-49 %
e. 50-69 %
f. 70-89 %
g. 90-100 %
h. Other:

3. With regard to treatment *(Med tanke på behandling)*

a. **Out of 10 dogs with diabetes, how many do you recommend a change of diet?** *(Av 10 hunder med diabetes, hvor mange endrer du diett på?)*

   i. None
   ii. 1
   iii. 2
   iv. 3
   v. 4
   vi. 5
   vii. 6
   viii. 7
   ix. 8
   x. 9
   xi. 10

b. **Out of 10 dogs with diabetes, how many do you recommend starting on subcutaneous insulin injections?** *(Av 10 hunder med diabetes, hvor mange startes på subkutane insulininjeksjoner?)*

   i. None
   ii. 1
   iii. 2
   iv. 3
   v. 4
   vi. 5
   vii. 6
   viii. 7
   ix. 8
   x. 9
   xi. 10
   xii. Other

c. **Out of 10 dogs with diabetes, how many do you start on peroral hypoglycaemic drugs?** *(Av 10 hunder med diabetes, hvor mange startes på perorale hypoglykemiske medisiner?)*

   i. None
   ii. 1
   iii. 2
   iv. 3
   v. 4
   vi. 5
   vii. 6
   viii. 7
   ix. 8
   x. 9
   xi. 10
   xii. Other

d. **Out of 10 intact female dogs with diabetes, how many are neutered?** *(Av 10 intakte tisper med diabetes, hvor mange kastreres?)*

   i. None
   ii. 1
   iii. 2
   iv. 3
   v. 4
   vi. 5
   vii. 6
   viii. 7
   ix. 8
   x. 9
   xi. 10
   xii. Other

e. **Of the female entire dogs that are NOT neutered, why are they not (multiple responses possible and write in an answer as needed under 'other')?** *(Av tisper med diabetes som IKKE kastreres, hvorfor kastreres de ikke (flere svar mulig, fyll inn under "other" ved behov)?)*

i. The owner does not want the bitch to undergo surgery *(Ønsker ikke at tispa skal opereres*)

ii. The owner does not recognise the importance of neutering (*Skjønner nok ikke viktigheten av kastrasjon*)

iii. Due to cost (*På grunn av kostnader*)

iv. The owner is worried about the overall quality of life of the dog with treatment, monitoring with the addition of surgery (*Eier er bekymret for livskvaliteten til hunden med både behandling og overvåking av diabetesen i tillegg til operasjon*)

4. Based on your experience; to what degree are owners of diabetic dogs concerned by *(Basert på hva du har erfart/mener; hvor* ***bekymret er eierne*** *til insulinbehandlede diabetikerhunder for)*

	Not worried	Mildly concerned	Moderately concerned	Very concerned	Not relevant
Hypoglycemia					
Diabetes ketoacidosis					
Having to inject their dog					
Costs associated with treatment					
The dogs' quality of life					
Owner having to make lifestyle changes					

Other:

5. Based on your experience treating diabetic dogs; how much are you concerned by (*Basert på hva du har erfart/mener; hva* ***bekymrer*** ***<u>deg</u>*** *mest med behandling av hunder med diabetes?)*

	Not worried	Mildly concerned	Moderately concerned	Very concerned	Not relevant
Hypoglycaemia					
Diabetes ketoacidosis					
The dogs' quality of life					
Difficulties acheiving fast and adequate control					
Difficulties getting owner on board or understanding what treatment entitles					
Costs associated with treatment					
My own competence in guiding the owner					
My own knowledge of diabetes mellitus					

Other:

6. Regarding the last two questions, are there any other concerns we have forgotten about? *(Med tanke på de to foregående spørsmålene; er det andre bekymringer vi har glemt?)*

a. Other:

OMITTED question: **Out of 10 dogs presenting to your practice, how many are euthanized at the time of diagnosis?** *(Av 10 hunder som presenterer til din praksis, hvor mange avlives ved diagnosetidspunkt?)*

a. 1
b. 2
c. 3
d. 4
e. 5
f. 6
g. 7
h. 8
i. 9
j. 10
k. Other:

7. Out of 10 dogs started on insulin injections in your practice, how many are euthanized within <u>one month</u> because of a lack of effect or compliance? *(Av 10 hunder som startes på insulininjeksjoner i din praksis, hvor mange avlives innen <u>en måned</u> pga. manglende effekt eller eierfaktorer?)*

l. 1
m. 2
n. 3
o. 4
p. 5
q. 6
r. 7
s. 8
t. 9
u. 10
v. Other:

8. Out of 10 dogs started on insulin injections in your practice, how many are euthanized within <u>one year</u> because of a lack of effect or compliance? *(Av 10 hunder som startes på insulininjeksjoner, i din praksis hvor mange avlives innen <u>ett år</u> pga. manglende effekt eller eierfaktorer?)*

a. 1
b. 2
c. 3
d. 4
e. 5
f. 6
g. 7
h. 8
i. 9
j. 10
k. Other:

9. Based on your experience, out of 10 dogs with newly diagnosed diabetes, how many achieve adequate QOL with insulin treatment? *(Basert på din erfaring, av 10 hunder med nydiagnostisert diabetes, ca. hvor mange oppnår tilfredsstillende livskvalitet <u>med</u> insulinbehandling?)*

a. 1
b. 2
c. 3
d. 4
e. 5
f. 6
g. 7
h. 8
i. 9
j. 10
k. Other:

10. Out of 10 *owners* of a dog with newly diagnosed diabetes, how many express that the insulin injections do *not* have too much of an effect on their lifestyle? *(Av 10 eiere til en hund med nydiagnostisert diabetes, hvor mange gir uttrykk for insulininjeksjonene ikke påvirker deres livsstil for mye?)*

a. 1
b. 2
c. 3
d. 4
e. 5
f. 6
g. 7
h. 8
i. 9
j. 10
k. Other:

11. On a scale from 1 to 10; are owners more likely to start treatment if their dog is insured? (1= no difference with or without. 10 = absolutely decisive for starting treatment). *(I en skala fra 1-10; er eiere mer tilbøyelige til å starte behandling dersom hunden deres er forsikret (1 = Ingen forskjell med eller uten forsikring. 10 = helt avgjørende for oppstart av behandling)?)*

a. 1
b. 2
c. 3
d. 4
e. 5
f. 6
g. 7
h. 8
i. 9
j. 10
k. Other:

12. On a scale from 1 to 10; are owners more likely to start treatment if themselves or people in their near social circle have diabetes? (1= no difference with or without. 10 = absolutely decisive for starting treatment) *(I en skala fra 1-10; er eiere mer tilbøyelige til å starte behandling dersom de SELV eller MENNESKER i NÆR omgangskrets har diabetes (1 = Ingen forskjell med eller uten diabetes i omgangskretsen. 10 = helt avgjørende for oppstart av behandling)?)*

a. 1
b. 2
c. 3
d. 4
e. 5
f. 6
g. 7
h. 8
i. 9
j. 10
k. Do not know
l. Other:

13. On a scale from 1 to 10; are owners more likely to start treatment if themselves or people in their near social circle have had a cat or dog with diabetes? (1= no difference with or without. 10 = absolutely decisive for starting treatment*) (I en skala fra 1-10; er eiere mer tilbøyelige til å starte behandling dersom de selv eller noen i nær omgangskrets har eller har hatt en KATT eller en HUND med diabetes (1 = Ingen forskjell med eller uten noen i omgangskretsen med hund/katt med diabetes. 10 = helt avgjørende for oppstart av behandling)?)*

a. 1
b. 2
c. 3
d. 4
e. 5
f. 6
g. 7
h. 8
i. 9
j. 10
k. Do not know
l. Other:

14. Would you have treated your own dog with insulin if it developed diabetes mellitus? *(Ville du ha behandlet din egen hund med insulin dersom den utviklet diabetes mellitus?)*

a. Definitely no
b. Maybe
c. Likely
d. Definitively yes
e. Other:

Monitoring

1. **In your practice, has an owner ever had their dog euthanised due to problems with monitoring their dog?** *(I din praksis, har en eier noen gang valgt å avlive hunden pga. problemer i forbindelse med overvåkning av diabetes?)*
    a. Yes
    b. No, never
    c. No, not only due to monitoring
    d. Other

2. **If yes, what form(s) of monitoring was used (multiple answers possible)?** *(Hvis ja; hvilken form for overvåkning var det (flere svaralternativer mulig)?)*
    a. At-home blood glucose curves
    b. In-hospital or in-clinic blood glucose curves
    c. Urine glucose
    d. Continuous interstitial glucose monitoring (e.g. FreeSyle Libre)
    e. Fructosamine measurements
    f. Other:

3. **If no, not solely due to monitoring, to what degree do you think monitoring affected the decision to euthanise?** *(Hvis nei, ikke kun pga. overvåkning: I hvilken grad medvirket overvåkningen til avgjørelsen om eutanasi for eier?)*
    a. No degree
    b. A small degree
    c. A middeling degree
    d. A moderate degree
    e. A significant degree
    f. Other

4. **What form of monitoring do you personally prefer in diabetic dogs (multiple answers possible)?** *(Hvilken form for overvåking foretrekker du hos pasienter med diabetes mellitus (flere svaralternativer mulig)?)*
    a. At-home blood glucose curves
    b. In-hospital or in-clinic blood glucose curves
    c. Urine glucose
    d. Continuous interstitial glucose monitoring (e.g. FreeSyle Libre)
    e. Fructosamine measurements
    f. Other:

5. **Have you ever used a continuous glucose monitor (CGM), e.g. Freestyle Libre)?** *(Har du brukt en kontinuerlig interstitiell glukosemåler (CGM) som for eksempel Freestyle Libre)?)*
    a. Yes
    b. No

6. **If yes** *(hvis ja)***:**
    a. **How is your experience with the use of Freestyle Libre (or equivalent) as a method of monitoring?** (*Hvordan er din opplevelse med bruken av Freestyle Libre (e.l.) som monitorerings- metode?)*
        i. Very positive
        ii. Somewhat positive
        iii. Neutral
        iv. Somewhat negative
        v. Very negative

b. **Comparing a CGM to other methods of monitoring at home or in clinic, what is POSITIVE with the CGM-use (multiple answers possible)?** *(Sammenliknet med annen overvåking hjemme eller på klinikken (blod eller urin), hva mener du er <u>positivt</u> med bruken av CGM (kan velge flere)?)*

  i. The dogs' quality of life improve and/or less stress due to a reduced number of needle pricks *(Hunden får bedre livskvalitet og/eller er mindre stresset ved å unngå stikk)*

  ii. More data is available for the assessment of treatment response *(At man får mer data til å vurdere behandlingen med)*

  iii. Owner seems less worried about diabetic complications such as hypoglycaemia, lack of insulin effect etc*. (Eier er mindre bekymret for komplikasjoner til diabetes som hypoglykemi, manglende effekt av insulin e.l.)*

  iv. Increases the communication between you as a vet and the owner, which again improves compliance. *(Øker kommunikasjonen mellom eier og deg som veterinær, som igjen gjør at man får en eier som følger rådene som gis)*

  v. Other positive experiences we have not mentioned? *(Evt. annet vi ikke har nevnt):*

c. **Comparing a CGM to other methods of monitoring at home or in clinic, what is NEGATIVE with the CGM-use (multiple answers possible)?** (*Sammenliknet med annen overvåking hjemme eller på klinikken (blod eller urin), hva mener du er* *<u>negativt</u>* *med bruken av CGM (kan velge flere)?)*

   i. Increased costs *(Økte kostnader i form av dyrere overvåkning)*
   ii. Owner gets hung-up on results and requires ver close follow-up *(Eier blir opphengt i resultater og krever veldig tett oppfølging)*
   iii. The owner gets hung up on results and changes the insulin treatment themselves *(Eier blir opphengt i resultater og endrer insulinbehandlingen selv)*
   iv. The owner seems more worried for complications, e.g. because of visualisation of changes in glucose *(Eier blir mer bekymret for komplikasjoner pga. visualisering av glukoseendringer)*
   v. Sensor functionality (e.g. stops working or falls off) is too short (*Varigheten til sensorene er for kort)*
   vi. Other negative experiences we have not mentioned? *(Evt. annet vi ikke har nevnt)*

d. **What is the most common reason(s) to a CGM-sensor must be changed before it has run out?** *(Hva er den eller de vanligste grunnen(e) til at en CGM-sensor må byttes (kan velge flere) før den er ferdigbrukt?)*

   i. The sensor falls off before 14 days has passed *(Sensoren faller av før 14 dager er gått)*
   ii. The sensor stopped working (error or faulty sensor) *Sensoren slutter å virke (feilmelding/feil med sensor)*
   iii. It was bothering the dog *(Den virker til å plage hunden)*
   iv. Inaccurate measurements *(Unøyaktige målinger)*
   v. The owner wants a new before the old has stopped working *(Eier ønsker ny før den gamle har sluttet å virke)*
   vi. Other experiences we have not mentioned? *(Evt. annet vi ikke har nevnt:*

e. **Do you change sensors yourself?** *(Bytter du sensor på selv på klinikken?)*

   i. Yes
   ii. No, I have taught a veterinary nurse or assistant to do it. (*Nei, jeg har lært opp dyrepleier/assistent til å gjøre det)*
   iii. No, I have taught the owner(s) how to do it *(Nei, jeg har lært opp eller lærer opp eier til å gjøre det)*
   iv. No, I send the patient to a colleague or another clinic for it. (*Nei, jeg sender det til en kollega/annen klinikk for dette*)

f. Based on your experiences, do you think it is stressful for the dog to have a new sensor placed*? (Basert på hva du har erfart/mener, virker det som at det er stressende for hunden å få satt på ny sensor?)*

   i. Yes
   ii. No
   iii. Sometimes
   iv. Other:

g. **Based on your experiences, how often do you see cutaneous reactions/changes in or around the sensor?** *(Basert på hva du har erfart/mener hvor ofte ser du reaksjoner i huden (rødme, hevelse, blødning, skorper, sår) under eller rundt sensoren til hunden?)*

   v. None
   vi. < 10%
   vii. 10-29 %
   viii. 30-49 %
   ix. 50-69 %
   x. 70-89 %
   xi. 90-100 %
   xii. Other:

h. **Do you use tissue glue to fasten the sensor?** *(Bruker du vevslim til å feste sensoren?)*

   i. Yes
   ii. No
   iii. Other:

i. **Based on your experiences, to what degree does cost play a role in the decision to place a CGM-sensor (e.g. a Freestyle Libre)?** *(Basert på hva du har erfart/mener, i hvilken grad har pris betydning for valget om å bruke en CGM-sensor (for eksempel Freestyle Libre) for eier?)*

   i. No degree
   ii. Some degree
   iii. Middeling degree
   iv. A moderat degree
   v. A very large degree
   vi. Other:

7. **If not used a CGM before, why not?** *(Hvis nei på CGM bruk, hvorfor ikke?):*
   a. Never heard of *(Aldri hørt om)*
   b. Novel technique I am not familiar with or comfortable using. *(Ny teknikk som jeg ikke kjenner/er komfortabel med å bruke)*
   c. Cost *(Kostnader)*
   d. Difficult to obtain sensors *(Vansker med å få tak i sensorer*)
   e. Other:

8. **If a subcutaneous interstitial continuous glucose monitor with a lifetime of 6-12 months was readily available in Norway (similar to an ID-chip). Would you be positive to use such a sensor?** *(Hvis det fantes en subkutan interstitiell glukosemonitor (tilsvarende en ID-chip) man kunne plassere under huden i 6-12 måneder. Er dette noe du kunne vært interessert i å prøve om det kom ut på markedet?)*
    i. Yes
    ii. No
    iii. Not sure
    iv. Other:

9. **If an owner were to be interested in a CGM (cutaneous or subcutaneous), what price range would you think the owners be willing to pay per month?** *(Hvis en eier skulle være interessert i en slik sensor, hvilken pris tror du eier hadde vært villig til å betale i måneden for et slikt produkt?)*
    i. 0-499 kr per month
    ii. 500-999 kr per month
    iii. 1000- 1499 kr per month
    iv. 1500- 1999 kr per month

Euthanasia

1. **What is your opinion of the prognosis of a diabetic dog***? (Hvor god eller dårlig prognose anser du at hunder med diabetes har?)*
    a. Prognosis is poor (*dårlig*)
    b. Prognosis is guarded to poor (*avventende til dårlig*)
    c. Prognosis is guarded (*avventende*)
    d. Prognosis is guarded to good (*avventende til god)*
    e. Prognosis is good (*god*)
    f. Other:
2. **In your experience, is the prognosis of diabetic female entire dogs better than female neutered or male dogs?** *(Er din oppfatning at diabetes mellitus hos intakte tisper har en bedre prognose enn hos kastrerte tisper eller hannhund?)*
    a. Yes
    b. No
    c. Do not know
    d. Other:
3. **Of the dogs you diagnose with diabetes mellitus, how many are euthanized primarily because of the diabetes?** *(Av hundene du diagnostiserer med diabetes; ca. hvor mange avlives som følge av sykdommen (diabetesen)?)*
    a. None (0%)
    b. 10% (9 of 10 dogs euthanised due to other causes)
    c. 20%
    d. 30%
    e. 40%
    f. 50%
    g. 60%
    h. 70%
    i. 80%
    j. 90%
    k. All (100%)
    l. Other:

4. Of the dogs euthanised, how many are euthanised due to owner wishes? *(Av hundene som avlives; hvor mange avlives etter ønske fra eier?)*

a. None (0%)
b. 10% (9/10 euthanised due to other factors)
c. 20%
d. 30%
e. 40%
f. 50%
g. 60%
h. 70%
i. 80%
j. 90%
k. All (100%)
l. Other:

5. The dogs euthanised as a result of owners' wishes, why do the owners want to euthanise? *(Med tanke på hundene som avlives etter ønske fra eier, hvorfor ønsker eier å avlive?)*

	Not important	Of little importance	Moderate importance	Of great importance	Not relevant
Cost (K*ostnader*)					
Quality of life (*Livskvalitet*)					
Owner's lifestyle alterations (*Eiers livsstilsendring*)					
Having to inject their dog (*Må injisere egen hund*)					
Monitoring issues (*Monitoreringsproblemer*)					
Prognosis (*Prognose*)					

Other:

6. Often, euthanasia is a result of a combination of the factors mentioned in the previous question, however which one do you think is the most important factor for the owners? *(Ofte vil avlivning ønskes som et resultat av en kombinasjon av faktorene oppgitt i forrige spørsmål. Av disse, hvilken tror du som veterinær veier tyngst for eierne?)*

a. Cost (*Kostnader*)
b. Quality of life (*Livskvalitet*)
c. Owners' lifestyle alteration (*Eiers livsstilsendring*)
d. Having to inject their dog (*Må injisere egen hund*)
e. Monitoring issues (*Monitoreringsproblemer*)
f. Prognosis (*Prognose*)

7. Of the dogs euthanised, how many are euthanised because of your recommendation? *(Av hundene som avlives, hvor mange avlives etter anbefaling fra deg?)*

a. None (0%)
b. 10% (9/10 euthanised due to other factors)
c. 20%
d. 30%
e. 40%
f. 50%
g. 60%
h. 70%
i. 80%
j. 90%
k. All (100%)
l. Other

8. With those dogs in mind that are euthanised due to your recommendation, how important are the following factors affecting your recommendation? *Med tanke på hunder som avlives etter anbefaling fra deg, hvor viktig er følgende faktorer anbefalingen din?*

	Not important	Of little importance	Moderate importance	Of great importance	Not relevant
Cost (*Kostnader*)					
Prognosis (*Prognose*)					
Comorbidities (K*omorbiditeter*)					
Complications (e.g. diabetes ketoacidosis) (K*omplikasjoner*)					
My own competence to guide the owner regarding monitoring and treatment (*Min egen kompetanse til å veiled eier med tanke på monitorering og behandling*)					
My own knowledge of diabetes mellitus, e.g. treatment and monitoring. (*Min egen diabetes kunnskap, feks om behandling og overvåking*)					

Other:

OMITTED QUESTION **Often, euthanasia is a result of a combination of the factors mentioned in the previous question, however which one is the most important factor for you?** *(Ofte er en anbefaling en kombinasjon av faktorene oppgitt i tabellen over, men av disse, hvilken veier tyngst for deg?)*

Demographics

1. **In what county do you practice (per 2024)?**
    a. Østfold
    b. Akershus
    c. Oslo
    d. Innlandet
    e. Vestfold
    f. Buskerud
    g. Telemark
    h. Agder
    i. Rogaland
    j. Vestland
    k. Møre og Romsdal
    l. Trøndelag
    m. Nordland
    n. Troms
    o. Finnmark
    p. Multiple, please fill out:
2. **My practice location is in** *(Min veterinærpraksis utføres i)*
    a. A large city (> 100.000 inhabitants) (*Storby > 100.000 innbyggere*)
    b. Suburbs (close to a large city) (*Forstad, nær stor by)*
    c. A village or rural area(s) (*Bygd eller landlige omgivelser*)
    d. A small city (> 5000, but < 100.000 inhabitants) (*liten by, mer enn 5000, men mindre enn 100.000 innbyggere)*
    e. Other:
3. **In my practice I see** *(jeg praktiserer)*
    a. Only small animals *(Kun smådyr)*
    b. Mixed animal practice *(Blandingspraksis)*
    c. Only seeing dogs out-of-hours *(Ser kun smådyr på vakt)*
    d. Other:
